# A first step in understanding an invasive weed through its genes: an EST analysis of invasive *Centaurea maculosa*

**DOI:** 10.1186/1471-2229-7-25

**Published:** 2007-05-24

**Authors:** Amanda K Broz, Corey D Broeckling, Ji He, Xinbin Dai, Patrick X Zhao, Jorge M Vivanco

**Affiliations:** 1 Center for Rhizosphere Biology, Colorado State University, Fort Collins, CO 80523-1173, USA; 2 Department of Horticulture and Landscape Architecture, Colorado State University, Fort Collins, CO 80523-1173, USA; 3 Cell and Molecular Biology Graduate Program, Colorado State University, Fort Collins, CO 80523-1173, USA; 4 Plant Biology Division, Samuel Roberts Noble Foundation, Ardmore, OK 73401, USA

## Abstract

**Background:**

The economic and biological implications of plant invasion are overwhelming; however, the processes by which plants become successful invaders are not well understood. Limited genetic resources are available for most invasive and weedy species, making it difficult to study molecular and genetic aspects that may be associated with invasion.

**Results:**

As an initial step towards understanding the molecular mechanisms by which plants become invasive, we have generated a normalized Expressed Sequence Tag (EST) library comprising seven invasive populations of *Centaurea maculosa*, an invasive aster in North America. Seventy-seven percent of the 4423 unique transcripts showed significant similarity to existing proteins in the NCBI database and could be grouped based on gene ontology assignments.

**Conclusion:**

The *C. maculosa *EST library represents an initial step towards looking at gene-specific expression in this species, and will pave the way for creation of other resources such as microarray chips that can help provide a view of global gene expression in invasive *C. maculosa *and its native counterparts. To our knowledge, this is the first published set of ESTs derived from an invasive weed that will be targeted to study invasive behavior. Understanding the genetic basis of evolution for increased invasiveness in exotic plants is critical to understanding the mechanisms through which exotic invasions occur.

## Background

Invasive weeds are regarded as major threats to biodiversity because they can spread through communities, displacing or even eradicating native species. Over 25,000 invasive plant species have been documented in the United States, invading nearly 700,000 hectares per year, with a cost exceeding 34.5 billion dollars per year [1]. Multiple non-exclusive ecological hypotheses exist to explain plant invasion in new habitats [2]. The niche hypothesis suggests invaders are able to take advantage of unutilized resources in new environments [3]. The natural enemy release hypothesis suggests invaders escape their natural enemies when moving to new environments, allowing them to obtain high population densities [4]. The novel weapons hypothesis suggests that invaders come equipped with an arsenal of chemical weapons that are detrimental to the resident community of the invaded habitat [5]. The plant community in the invader's native habitat has had time to co-evolve defenses against these chemical weapons, whereas the invaded community has not, allowing invaders to obtain a competitive advantage in their new environment [5]. The evolution of increased competitive ability (EICA) hypothesis expands on the idea of natural enemy release, suggesting that invaders have rapidly evolved in their new environment to direct more resources to competitive ability over defense [6]. Similarly, the allelopathic advantages against resident species (AARS) hypothesis expands on the idea of novel weapons, suggesting that novel weapons which increase plant competitive ability are selected for in the invasive range [7]. All these hypotheses are supported at least in part by data from field experiments which are often coupled with physiological and biochemical studies; however, it remains unclear why some plants become problematic invaders and others do not. One aspect that is rarely investigated in relation to invasive weeds and their native counterparts is the potential for modified gene regulation in the introduced range. As limited genetic resources are available for most invaders and other weedy species [8], defense and growth response genes cannot be effectively monitored at the molecular level to test hypotheses of plant invasion.

*Centaurea maculosa *Lam. (spotted knapweed) is a Eurasian native that has become a particularly problematic invasive weed in the northwestern United States, infesting over 4.5 million acres in Montana alone [9]. Spotted knapweed often colonizes disturbed areas in North America, but also invades rangelands, pastures and prairies, where it displaces native species and establishes dense monocultures. Diploid and tetraploid forms of the weed exist in the native range, but only tetraploid plants have been identified in North America [10]. The diploid form contains 18 chromosomes [11], with a DNA content (2C) near 3.6 picograms based on measures from closely related species of the same chromosome number [12]. This translates to an estimated genome size of approximately 1,800 Mbp. Molecular markers and karyotyping have been used to identify the two forms, as it is impossible to distinguish between them based on morphological characters [10]. Both forms are short-lived outcrossing perennials of the aster family; however, the diploid is monocarpic while the tetraploid is polycarpic. Also, the tetraploid can tolerate dense vegetation and thus may be a better competitor than the diploid. This is presumed to be the main reason why only the tetraploid form is invasive in North America, while it is not considered invasive or even predominant in its native Eurasian habitat [10]. Ecological and greenhouse investigations suggest that invasive *C. maculosa *is a strong competitor against North American natives, even in the presence of biocontrol agents that have been introduced to limit the spread of the weed [13-17]. Also, common garden studies suggest that North American *C. maculosa *seeds germinate more readily than their Eurasian counterparts and the resulting individuals are larger, more robust, and better able to fend off and compensate for herbivore attack, under both greenhouse and field conditions [Ridenour *et al*. submitted]. These studies give partial support to the EICA hypotheses. There is also evidence that *Centaurea *species (*C. maculosa *and *C. diffusa*) produce allelopathic compounds that inhibit growth and germination of North American plant species more severely than their native congneners, lending support to the novel weapons hypothesis [5,7,15,16], although the production of allelochemicals seems to be variable [18]. It is not clear what combination of effects cause *C. maculosa *to become invasive in North America.

As an initial step towards understanding the molecular mechanisms by which plants become invasive, we have generated a normalized Expressed Sequence Tag (EST) library representing seven invasive populations of *C. maculosa*. Here we describe candidate genes that could be utilized in future experiments to correlate plant gene expression and ecological hypotheses proposed for invasive success. To the best of our knowledge, this is the first published set of ESTs derived from an invasive weed that will be targeted to study invasive behavior.

## Results and discussion

### Library creation and sequence annotation

Seeds from seven invasive populations of *Centaurea maculosa *Lam were grown for one to two months, at which time two to three entire plants per population were harvested. Tissue was frozen in liquid nitrogen and shipped to Agencourt biosciences for normalized cDNA library construction. Single pass directional sequencing was performed on 4969 randomly selected clones from the *C. maculosa *normalized cDNA library (GenBank accessions EL930664-EL935630). These sequences were assembled into 4423 unique contigs (contiguous consensus sequences) or "unigenes" using Noble Foundation's in-house pipeline based on TIGR Assembler [19].

The library consisted of 894 contig-forming ESTs which created 348 unigenes and 4075 singlet ESTs, each representing a unique sequence. Of the 348 unigene contigs, the majority contained only two EST sequences [see Additional File 1]. The largest unigene contig, which had high similarity to a chlorophyll a-b binding protein, contained 17 ESTs. Interestingly, the second largest unigene contig, a compilation of ten sequences, had extremely low similarity to known sequences and was not able to be annotated. Other large unigene contigs were annotated as polyphenol (catechol) oxidase, chloroplast ATP synthase, photosystem I subunits II and XI, polygalacturonase (a pectinase), and the small subunit of RUBISCO. Normalization should remove most redundant transcripts and enrich for low abundant regulatory genes in the library. However, it is interesting that one of the most abundant transcripts found, polyphenol oxidase, is a potential defense-related protein [20]. Overall redundancy of the library was 18% (number of clustered ESTs/total ESTs) which suggests that the normalization process was effective, and that continued sequencing of the library has the potential to uncover many more unique transcripts. Sequence quality was high; over 80% of the unigenes were between 800–1100 bp in length [see Additional File 2], with an average size of 784 bp.

To annotate the *C. maculosa *ESTs, the 4423 unigenes were translated in all frames and searched for similarity against the NCBI non-redundant protein database using BLASTX (E-value of 10^-4 ^or less). Of the entire unigene set, 77% (3392) had significant similarity to genes in the NCBI database, while the remaining 1031 sequences had low similarity and were not able to be annotated. In the group of annotated sequences, 35% (1177 unigenes) had top BLAST hits to transcripts from *Arabidopsis thaliana*, whereas only 10% (338 unigenes) had top hits to *Orzya *cultivars. Taxonomically, 64% (2182) of the annotated unigene top hits grouped into the rosids clade (which includes the families Brassicacea and Fabaceae), 20.6% grouped into the asterids clade (which includes the families Asteraceae and Solanacea), and 11.8% grouped into the commelinids clade (which includes members of the Poaceae family and other monocots) [see Additional File 3]. Thirty four unigenes had top hits to non-plant sequences. Most of these non-plant sequences were annotated as hypothetical or unknown proteins.

### Functional categorization of Centaurea unigenes

**Figure 1:**
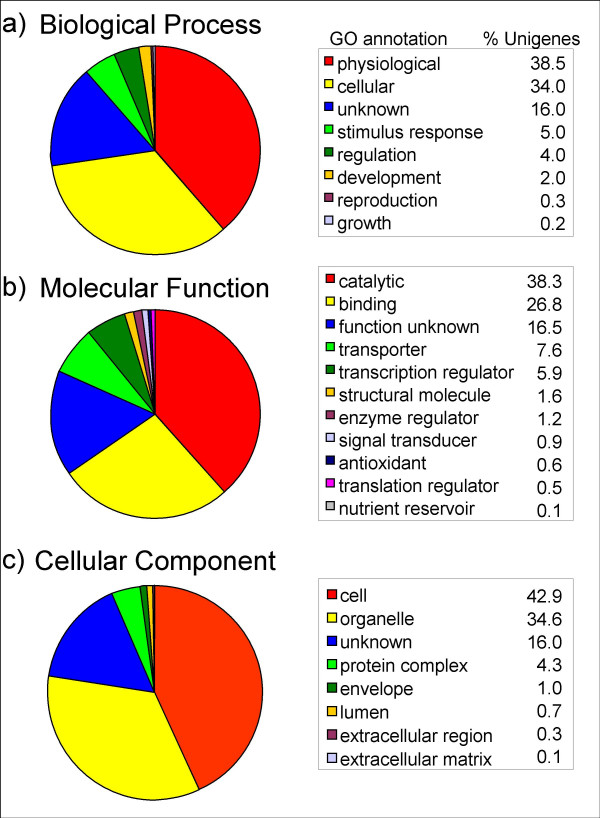
**Gene Ontology annotation of *Centaurea *Unigenes**. A normalized cDNA library was created from whole plants representing seven invasive populations of *Centaurea maculosa*. Five thousand ESTs were sequenced from the 5' end (Agencourt biosciences), and assembled into 4,423 contigs, or '*Centaurea *unigenes.' Unigenes were translated in all frames and the resulting amino acid sequences were used as BLAST queries. Top BLAST hits provided annotation and functional categorization (gene ontology assignment) for each *Centaurea *unigene. Not all unigenes were able to be annotated by GO programs. Computational analysis was done using the PLAN database (Noble Foundation).

Gene ontology (GO) assignment programs were used to functionally categorize unigenes in the library. Unigene GO terms were counted and grouped in a hierarchical fashion into the major GO functional categories of Cellular Component, Biological Process and Molecular Function. The GO categories of cellular component and biological process contained over 3000 *Centaurea *unigene annotations, whereas the molecular function category contained only 2162 annotations. Each category contained approximately 16% unigenes that were annotated as "unknown," but this number does not account for the unigenes that were unable to be annotated in GO format (Figure 1).

Approximately 38% of the unigene annotations were grouped into the 'physiological process' category of the Biological Process GO (Figure 1), which includes subcategories such as metabolism, transport, photosynthesis, apoptosis, and homeostasis. The next largest category of unigene annotations (~34%), 'cellular process,' has some overlap of subcategories with physiological processes (i.e., metabolism, transport), but includes unique subcategories such as cell communication, recognition, and differentiation. Five percent of unigene annotations fell in the 'stimulus response' category which includes subcategories that relate to plant response to abiotic and biotic stresses, such as pathogen attack. Thirty unigenes were subcategorized as responding to hormone stimulus, and most of these fell into the ethylene and jasmonic acid signaling pathways. Ethylene- and jasmonic acid-mediated pathways have been implicated in the defense of plants against pathogens and insects [21,22], and these transcripts may be up-regulated in *C. maculosa *under biotic stress conditions. Only a small percentage of unigene annotations fell into the reproduction category of biological processes (0.3%), but this is not entirely surprising, as reproductive structures such as flowers were not used in the starting material for the library.

Over 38% (829) of the unigene annotations fell into the 'catalytic activity' category of the molecular function GO (Figure 1). Specific catalytic activities associated with these unigenes covered a range of GOs, with the largest amount of unigenes falling into the transferase and hydrolase categories (282 and 266 unigenes, respectively). These types of enzymes are involved in many intracellular processes including primary and secondary metabolism, signal transduction, and post translational modification of proteins. The next largest category under molecular function was 'binding' (~27%, 580 unigenes) with the majority of unigenes being associated with nucleotide/nucleic acid binding (134 and 230 unigenes, respectively).

Transporters accounted for nearly 8% of all unigene annotations, the majority being ion transporters and transporters with carrier activity (54 and 48 unigenes). Eighteen unigenes fell into the 'ATP-ase coupled transporter' category, which includes transporters of xenobiotics, steroids, sugars, peptides and other small molecules. However, most unigenes could not be subcategorized with a specific transport role. Uptake and translocation of nutrients in plants differ, and regulated expression of specific transporters may allow increased competitive ability in different situations (e.g., increased expression of metal transporters may be beneficial in metal-limiting environments) [23-25]. Depending on the specific transporter, these genes could be interesting targets for understanding root exudation or other release strategies for *Centaurea *secondary metabolites and uptake of nutrients, and may aid in understanding the role of transport in competitive ability of *Centaurea*.

One hundred twenty-seven unigene annotations were designated as transcriptional regulators, with the majority (118) being subcategorized as having 'transcription factor activity.' Transcription factors are responsible for modulating cellular responses to biotic and abiotic stimuli [26], and they may play important roles in plant invasion by up- or down-regulating the expression of genes involved in defense and growth responses. Transcription factors identified in the *Centaurea *library made up ~6% of the GO annotations for molecular function, whereas ~4% of the *Arabidopsis *genome sequences are annotated as transcription factors (The *Arabidopsis *Information Resource, TAIR).

Over two-thirds of the unigenes were localized by cellular component to either cell or organelle, as shown in Figure 1. Of these, 164 unigenes were assigned to the nucleus, 357 to the mitochondrion, 52 to the chloroplast, and 37 to the cytosol. Thirty-five unigenes were assigned to the cell wall category and 290 unigenes were assigned to the membrane category, although only 27 could be further categorized to the plasma membrane. These membrane proteins may be interesting targets to investigate transport and/or signal transduction.

A wide variety of functional categories were represented in the *Centaurea *library. Many of these unigenes could be used as candidates for production of a microarray to visualize changes in global gene expression, or to look at more specific changes in regulation related to plant defense or stress.

### Unigene candidates for testing ecological hypotheses

#### Evolution and plasticity

The EICA hypothesis suggests that when plants are introduced into a new range, they escape their enemies and rapidly evolve to put more resources into growth/reproduction and less into defense [5]. Evolution through random mutation, movement of transposable elements, and genetic recombination may facilitate changes in plant genes or gene expression which give them a competitive and evolutionary advantage [27]. In addition, novel environments can reveal genetic variants in a population that possess advantageous phenotypes due to adaptive or developmental plasticity [28]. Candidate unigenes from the *Centaurea *cDNA library potentially involved in genome evolution and plasticity are described below.

##### Mobile elements

Mobile, or transposable elements (transposons) have the ability to modify DNA sequences by 'jumping' in and out of places in the genome, and certain mobile elements carry gene fragments that, when transposed, lead to repetition or creation of new genetic material [29]. Mobile elements can modify genome size, gene regulation, and gene function, all of which contribute to genome evolution [29,30]. In the invasive *Centaurea *cDNA library, six transposable element-related unigenes were identified [see Additional File 4]. Normally transposable elements are found in non-coding regions of their host genomes and are considered 'silent;' however, expressed transposable elements, such as those found in the *Centaurea *cDNA library, have been detected in plants at specific growth stages and under biotic stress conditions such as pathogen attack and wounding [32,33]. Evidence from such experiments support Barbara McClintock's idea that transposable elements may play a role in genome evolution through organismal adaptation to stress [34], and it would be interesting to test this idea in relation to plant invasion.

##### Heat-shock proteins

Heat shock protein 90 (Hsp90), a stress-induced protein, has been shown to buffer genetic variation in morphogenic pathways in the fruit fly *Drosophila melanogaster *and the cruciferous plant *Arabiodpsis *[28]. As a chaperone of proteins that regulate growth and development, Hsp90 may allow for the storage and release of genetic variation, as well as allowing phenotypic plasticity in an organism's response to their environment [28,35]. Eight heat shock-related unigenes were identified in the *Centaruea *cDNA library [see Additional File 4]. One unigene is closely related to Hsp90, one to Hsp60, one to Hsp70, and the others are annotated as 'putative heat-shock proteins' or 'heat shock factors.' Only two of these unigenes have top BLAST hits to sequences from *A. thaliana*, suggesting there may be a wide diversity of Hsp90 related sequences in *Centaurea*. Understanding the mechanism of these proteins in relation to plant genotype, environment and defense response throughout the native and invasive range of *C. maculosa *may give some clues to the plasticity of the species.

#### Secondary metabolism

As explained above the AARS hypothesis proposes that novel weapons, often in the form of secondary metabolites, are selected for in invasive plants in the introduced ranges. Members of the aster family are capable of synthesis of a broad spectrum of secondary metabolites that may aid in basal and induced defense response, as well as in competition against other plants [16,36]. Included in the list of secondary metabolites synthesized by *Centaurea spp*. are polyacetylenes and related thiophenes, flavonoids (flavones and flavonols and their derivatives in particular) and their glycosides, phenolics and lignans, coumarins, anthocyanins, cyanogenic glycosides (prunasin), mono-, sesqui-, di- and tri-terpenoids (with sesquiterpene lactones particularly diverse), and steroidal compounds [36]. Described below are several *Centaurea *unigenes which share sequence similarity with characterized genes involved in plant secondary metabolite biosynthesis. Further study of these candidate genes may aid in understanding the relative influence of AARS in *Centaurea *invasion.

##### Sesquiterpene lactones

*C. maculosa *is known to accumulate the sesquiterpene lactone, cnicin, at concentrations approaching 2% of dry weight on the leaves of the inflorescence stem [37]. This compound is thought to act as a protectant against herbivory of generalist herbivores and acts as an oviposition stimulant for specialist herbivores [38] including *Agapeta zoegana*, a biological control agent introduced for the control of *C. maculosa *in the North America. Additionally, cnicin is phytotoxic to several plant species [39], appears to inhibit the rumen microbial activity of sheep (thereby reducing digestibility of *C. maculosa *[40]), possesses broad spectrum anti-fungal activity [41], and is being examined as a potential pesticide for control of formosan termite [42]. Sesquiterpenes are synthesized through cyclization of farnesyl pyrophosphate (FPP) followed by further modification steps including oxidation, reduction, and glycosylation reactions. The genes responsible for the committed step that catalyzes the conversion of FPP to sesquiterpene hydrocarbons are well-characterized, including sesquiterpene synthases from aster family members such as *Artemisia annua*, *Solidago canadensis*, *Helianthus annuus*, *Ixeris dentata*, *Chicorium intybus*, and *Lactuca sativa*. In the *Centaurea *cDNA library, no unigenes were annotated as a sesquiterpene synthase. However, several unigenes revealed high sequence simlarity to genes involved in the synthesis of the iosprenoid pyrophosphates, metabolic precursors to the terpenoids. BLAST similarity searches revealed two unigenes which closely matched the *Antirrhinum majus *geranyl diphosphate synthase, which catalyzes the condensation of two isopenyl pyrophosphate units to form a ten-carbon precursor (geranyl pyrophosphate – GPP) of monoterpenoids. GPP can then be extended further to FPP, geranylgeranyl pyrophosphate (GGPP), and ultimately to dolichol phosphate, a polyprenoid involved in the formation of glycoproteins via the endomembrane system. Two unigenes were annotated as dehydrodolichol phosphate synthases by GO annotation.

Following formation of the ring structure, sesquiterpene skeletons can be modified through oxidation, reduction, and glycosylation reactions to form an enormous diversity of secondary products including cnicin, a sesquiterpene lactone found in *C. maculosa*. Sesquiterpene lactones commonly occur in the Asteraceae, but the biosynthetic routes for individual metabolites are relatively poorly characterized. One of the most well-characterized of the biosynthetic pathways leading to sesquiterpene lactones is that for artemisinin, a compound of value as an anti-malarial drug [43] isolated from the aster *Artemisia annua*. Recently the oxidation steps which generate artemisinic acid, a precursor of artemisinin, were characterized and the entire pathway to artemisinic acid reconstructed in yeast [44]. The reaction proceeds from FPP to the sesquiterpene, amorphadiene (catalyzed by amorphadiene synthase), and then to artemisinic acid, a reaction catalyzed by a single p450 enzyme which performs a three-step oxidation reaction to form a carboxylic acid. A unigene from the *Centaurea *library (CENT_UG_03500) demonstrates 93% amino acid sequence identity to this three-step oxidase and a nearly identical gene from the related *A. obtusifolia *[see Additional File 5], highly suggestive of a role in the biosynthesis of the cnicin or other sesquiterpene lactones of *C. maculosa*.

##### Acetylenes

**Figure 2:**
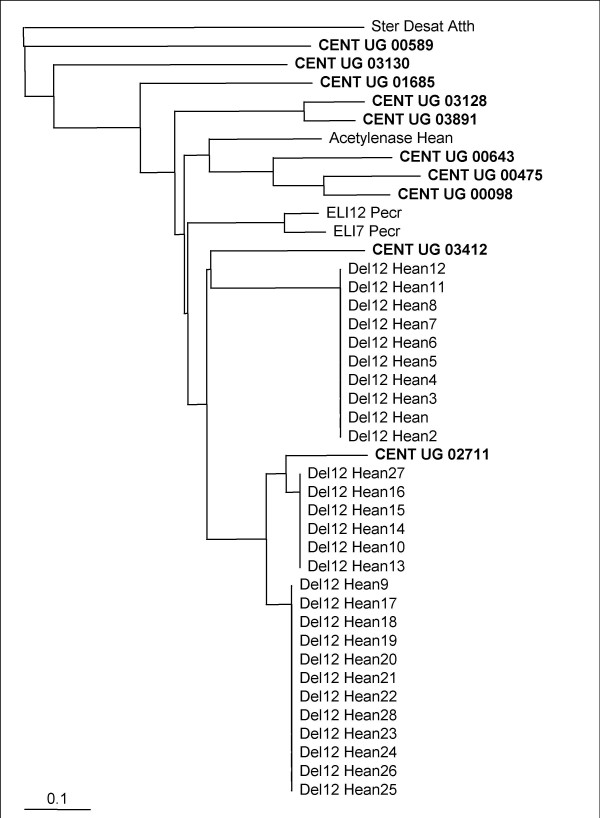
**Phylogenetic analysis of acetylenease-related sequences from *Centaurea *cDNA library**. *C. maculosa *EST sequences with similarity to fatty acid desaturase genes were clustered with previously characterized genes from *Helianthus annuus *(Hean), *Petroselinum crispum *(Pecr) fungal elicited desaturases (ELI), and with sterol desaturasase from *Arabidopsis thalania *as an outgroup. CENT_UG_00643, CENT_UG_00475, and CENT_UG_00098 cluster with the characterized acetylenase from *H. annuus*, distinct from the remaining Δ12 desaturases (Del12), suggesting potential for acetylenase activity.

The polyacetylelenes are secondary metabolites derived from fatty acids, and are characteristic of many aster genera. Acetylenes contain highly reduced carbon-carbon triple bonds. The biosynthetic route has been only recently characterized for a small group of acetylenes [45]. Alignment of established acetylenases, Δ^12 ^oleic acid desaturates, and *C. maculosa *unigenes annotated as desaturases reveals three *C. maculosa *ESTs which cluster closely to *H. annuus *acetylenase, but are distinct from *H. annuus*Δ^12 ^oleic acid desaturates (Figure 2), suggesting that these genes may be involved in acetylene production in *C. maculosa*.

##### Flavonoids

The basic flavonoid pathway is the best-characterized metabolic pathway of plant secondary metabolism [46]. More than 800 flavonoid structures have been characterized from the Asteraceae, in the Cardueae tribe, which contains *Centaurea spp*., that are particularly rich in hydroxy-methylated flavonols and flavones [47]. The early steps of the pathway involve the generation of phenylpropanoid monomers which are condensed with three malonyl CoA units to form the chalcones, followed by isomerization, hydroxylation, methylation, glycosylation, and polymerizations steps [46]. Many steps of the general flavonoid pathway are represented in the *Centaurea *cDNA library, with clones present that show similarity to most characterized enzymatic functions [see Additional File 6]. However, no unigenes showed significant sequence similarity to flavonol synthase (FLS), the gene responsible for generation of the flavonols, or flavone synthase (FS), the gene product of which converts flavanones to flavones. Each of these genes are oxoglutarate-dependent dioxygenases, and several unigenes suggest this function, as revealed by GO annotation. Further, only two FS clones from Asteraceae members (*Gerbera hydrida *and *Callistephus chinensis*) are reported in GenBank, and only one Asteraceae sequence is annotated as an FLS gene (from *G. hybrida*), suggesting that one or more of the unannotated 2-oxoglutarate-dependent dioxygenases may be FLS or FS genes.

As *C. maculosa *has been reported to exude catechin, a phytotoxic secondary metabolite, from its root [15,16], the study of genes involved in secondary metabolism in *C. maculosa *may help reveal how this compound and other potential 'novel weapons' are synthesized. The AARS hypothesis suggests that plants may evolve to produce more of the effective 'weapon' compounds when in the invaded environment [7], so it would also be interesting to test the activity of some of these proteins in native and invasive populations.

#### Defense-response genes

One of the main predictions of EICA is that plants in their new environment rapidly evolve to put more resources into growth/reproduction and less into defense, as they have escaped their co-evolved pathogens and predators [5]. Thus, native plants should show higher levels of basal defense compounds and should out-perform invasives when both are exposed to pathogens from the native environment. The *Centaurea *cDNA library contains a variety of unigenes annotated as defense response genes [see Additional File 7], as well as components of signaling pathways that may be involved in defense mechanisms. These include *Centaurea *sequences similar to three lipoxygenase (LOX) proteins, two phenylalanine ammonia lyase (PAL) proteins, and three calmodulin binding proteins. Also identified was a *Centaurea *sequence similar to the *Arabidopsis *activated disease resistance-like (ADR1-like) gene, which contains nucleotide binding site – leucine rich repeat (NBS-LRR) motifs characteristic of defense-response proteins [48]. Four *Centaurea *unigenes show high similarity to another LRR containing *Arabidopsis *transcript (At3g20820) that is suspected to be involved in the defense response signal transduction pathway. The *Centaurea *library contains seven other unigenes, not annotated as "defense related," that contain LRR motifs or leucine zippers [see Additional File 7]. One *Centaurea *unigene shows similarity to the R gene-mediated disease resistance gene (EDS1) from *Arabidopsis*, which is required for SA accumulation and production of pathogenesis related (PR) proteins [49]. Two unigenes show similarity to a PR-1 type protein from *Sambucus nigra*, similar to *Arabidopsis *PR-1-related transcript At4g33720. In addition, twelve of the unigenes annotated as having 'transcription factor activity' in the GO molecular function categorization are similar to WRKY transcription factors [see Additional File 7] which may be involved in initial steps of the defense-response signaling pathway [50]. With this *C. maculosa *sequence information it will be possible to test levels of basal defense responses at the level of gene expression in populations of native and invasive plants. Genes involved only in induced defense response may be under represented in the library, as the plants that were used in library creation did not undergo interspecific competition, pathogen stress, or herbivory and were not grown under field conditions. However, many of the unigenes identified have been implicated in induced defenses in other systems (PR-1 and EDS-like unigenes), and these may be good candidates for studying induced as well as basal defense response. Future experiments can be coupled with more traditional tests of morphology and biochemistry in order to supplement data concerning hypotheses of plant invasion.

## Conclusion

This is the first report of a cDNA library from an invasive weed. The *Centaurea *cDNA library, consisting of 4423 unique transcripts (unigenes), represents an initial step towards looking at gene-specific expression in this species, and will pave the way for creation of other resources such as microarray chips that can help provide a view of global gene expression in invasive *C. maculosa *and its native counterparts. These technologies can likely be extrapolated to look at other invasive knapweeds (*C. diffusa, C. solstitialis, C. virgata *and *Acroptilon repens*) also problematic in North America. By comparing native and invasive *C. maculosa *plants under different stresses, including herbivory and pathogen infection, it will be possible to test hypotheses such as EICA using molecular resources coupled with classical (physiological/ecological) techniques.

This technology will also be useful to help understand differences in gene expression between diploid and tetraploid *C. maculosa *populations, and give insight into the effects of chromosome doubling and polyploidization events in the plant world. Additionally, by looking at secondary metabolite accumulation and the genes responsible for their production in *C. maculosa*, it may be possible to knock out those genes, create mutants defective in the production of allelochemicals, and to finally determine unequivocally whether allelopathy (novel weapons) is involved in the invasive success of some weeds.

Understanding the genetic basis of evolution for increased invasiveness in exotic plants is critical to understanding the mechanisms through which exotic invasions occur. The *Centaurea *cDNA library provides a unique resource that will be valuable to geneticists, molecular biologists, and ecologists alike.

## Methods

### Plant material

Seeds from seven invasive populations of *Centaurea maculosa *Lam were obtained from Ray Callaway (University of Montana, Missoula). Five populations originated from Montana, one from Washington, and one from Virginia. Six seeds from each of the seven populations were sterilized by heating at 50°C for ten minutes in distilled water. Seeds were cooled to ambient temperature, rinsed with sterile water and placed in Petri dishes containing moist germination paper. Plates were wrapped in parafilm and placed in a growth chamber with a photoperiod of 20 hours light/six hours dark at a constant temperature of 25°C. Upon the emergence of cotyledons, seedlings were planted in 2.5 cm pots in a mix of 70% sand, 10% perlite, and 20% autoclaved potting soil and transported to the greenhouse. Pots were placed in a flat and covered with plastic wrap for approximately one week until seedlings became established. Plants were given sufficient water and fertilized once per week with a dilute solution of Miracle Gro (Maryville, OH). After approximately two months, two to three plants per population were removed from pots and their roots were washed to remove soil particles. All plants were in the form of small rosettes, lacking stem and floral tissue. Entire plants, including roots, were wrapped in foil, frozen in liquid nitrogen, and stored at -80°C until processing.

### Creation of normalized cDNA library

Plant tissue was shipped in dry ice to Agencourt Bioscience Corporation (Beverly MA). Total RNA was extracted and optimized first strand cDNA synthesis was performed using a primer adapted with a rare enzyme cut site. cDNA fragments were size-selected by agarose gel electrophoresis, and directionally cloned into a pAGEN-1 vector. A positive control containing the Tet^R ^gene was used during construction of the primary library to ensure library quality. Single-stranded DNA was made from a portion of the primary library by phagemid production, and reactions were treated with DNase I to ensure the removal of double-stranded DNA. A second portion of the primary library was linearized and transcribed into anti-sense RNA with biotinylated dNTPs. Oligo dT and primer extension were used to pre-block the poly-A region prior to hybridization. The anti-sense RNA and single-stranded circular DNA were hybridized, and abundant clones were removed using streptavidin. To reduce the amount of empty vectors, a Not1 oligo and *Taq *polymerase were used to synthesize double stranded DNA from the single stranded normalized library prior to final transfection.

The normalized library was plated and 4969 clones were randomly selected for sequencing. Automated plasmid purification was achieved using the SPRI (SprintPrep™) technique, which harvests plasmid DNA directly from lysed bacterial cultures, trapping both plasmid and genomic DNA to functionalized bead particles and selectively eluting only the plasmid (Beckman Biomek FX robots and CCS Packard DNATraks).

### Sequencing reactions

DNA templates were sequenced in 384-well format using BigDye^® ^Version 3.1 reactions on ABI3730 instruments at Agencourt Biosciences. Thermal cycling was performed using 384-well Thermal cyclers (ABI, MJ Research). Sequencing Reactions were purified using Agencourt's CleanSeq^® ^dye-terminator removal kit. All reads are processed using Phred base calling software and constantly monitored against quality metrics using the Phred Q20. The quality scores for each run were monitored through the Oracle 9i driven Laboratory Information Management System (LIMS). *C. maculosa *ESTs were trimmed of vector sequence and the data was transferred to a secure site for download.

### Sequence analysis

To determine the number of unique transcripts in the library, an in-house pipeline program was used to cluster and assemble the trimmed EST sequences. The pipeline essentially utilizes TIGR Assembler with its default parameters (overlap of at least 40 bp with 94% identity). The PLAN web system (Personal BLAST Navigator, Noble foundation) was used to do a BLASTX search against the non-redundant protein (NR) database for functional annotation, and gene ontology (GO) sequence database for functional categorization [19]. The BLASTX search considered translation of the assembled consensus (unigenes) in multiple reading frames. The top NR hit for each unigene sequence (E-value 10^-4 ^or less) and top hits from GO assignment were deposited in PLAN and can be searched by keyword or unigene accession number (PLAN Project 30060, CENT_UG_00001-CENT_UG_04423). GO annotation was used to categorize unigenes into functional categories by molecular function, cellular component and biological process. A customized in-house program was used to count the number of unigenes being grouped under different GO term categories in a hierarchical fashion [19]. The vector trimmed EST sequences have also been deposited in GenBank (accession numbers EL930664-EL935630).

## Authors' contributions

AKB: designed and performed research, analyzed data, wrote manuscript

CDB: analyzed data, wrote manuscript

JH, XD, PXZ: provided new analytical techniques (PLAN database)

JMV: designed research, edited and approved manuscript

## Data deposition

GenBank Accession numbers:

EL930664-EL935630

Database EST ID (NCBI) numbers:

45411770-45416736

PLAN database accession numbers for Centaurea EST sequences:

(public projects section, project 30060)

CENT_UG_00001 through CENT_UG_04423

## Supplementary Material

Additional file 1 **Distribution of assembled *Centaurea *ESTs by cluster size**. The data represent clustering of *Centaurea *ESTs into unique sequence clusters (unigenes) and show distribution by cluster size. The 4969 *Centaurea *ESTs were assembled into 4423 unique contigs or 'unigenes' using the PLAN database (Noble foundation). In total, 4075 singlet ESTs were unique (not pictured on graph); 348 could be assembled into clusters containing one or more *Centaurea *unigene, and were plotted relative to their abundance in the EST library. Click here for file

Additional file 2 **Title: Distribution of *Centaurea *unigenes by sequence length**. The data represent distribution of *Centaurea *unigenes by sequence length. The 4423 *Centaurea *unigenes were plotted by their relative abundance based on sequence length in base pairs. Click here for file

Additional file 3 **Taxonomic clades associated with *Centaurea *unigene top BLAST hits**. The data represent taxonomic clades associated with the top similarities of known sequences to *Centaurea *unigenes. *Centaurea *unigenes were used to query BLASTX nr database, and the top hit for each unigene was deposited in the PLAN database (3392 unigenes had significant top BLAST hits, others were unable to be annotated). These top hits were assembled by taxonomic group. Approximately 35% of the unigenes had top hits to *Arabidopsis*, which is part of the Rosid clade. Click here for file

Additional file 4 **Evolution/Plasticity-related sequences in *Centaurea *cDNA library**. The table lists sequences identified in the *Centaurea *cDNA library that may be related to evolution and plasticity, based on similarity to known sequences. Evolution-plasticity related sequences from the *Centaurea *cDNA library are represented by *Centaurea *unigene identification number (from the PLAN database). Accession number, organism, functional description, and E value of the top BLAST hit for each unigene is listed. Click here for file

Additional file 5 **Alignment of *Centaurea *unigene (CENT_UG_03500) and related sequences**. The data represent predicted amino acid sequence alignment of *Centaurea *unigene 03500 with related sequences involved in sesquiterpene lactone synthesis. Sesquiterpene lactone synthesis proteins from *Artemisia obtusifolia *and *A. annua *were aligned with *Centaurea *unigene 03500 using (Clustal W). Stars (*) indicate complete sequence conservation;**(:) **represents amino acids of a similar nature. Click here for file

Additional file 6 **Flavanoid Pathway- related sequences in *Centaurea *cDNA library**. The table lists sequences identified in the *Centaurea *cDNA library that may be involved in the flavanoid pathway, based on similarity to known sequences. Proposed function (Func) of flavanoid pathway related sequences in the *Centaurea *cDNA library; PAL (phenylalanine ammonia lyase), C4H(cinnamate 4-hydroxylase), C4L (4-coumaryl-CoA ligase), CHS (chalcone synthase), CHI (chalcone isomerase), F3'H (flavanoid 3'-hydroxylase), GT (glycosyl transferase), OMT (O-methyltransferase). The number of unigenes and their identification numbers (PLAN database) are listed for each functional group. Click here for file

Additional file 7 **Defense-response-related sequences in *Centuarea *cDNA library**. The table lists sequences identified in the *Centaurea *cDNA library that may be involved in defense response, based on similarity to known sequences. Defense-response-related sequences from the *Centaurea *cDNA library are represented by *Centaurea *unigene identification number (PLAN database). Accession number, organism, functional description, and E value of the top BLAST hit for each unigene is listed. Click here for file
