# Supplementary material for: A first step in understanding an invasive weed through its genes: an EST analysis of invasive Centaurea maculosa
Source: BMC Plant Biol. 2007 May 24;7:25. doi: 10.1186/1471-2229-7-25 (PMC1890287; doi:10.1186/1471-2229-7-25)
Supplement: Additional file 1 — Distribution of assembled Centaurea ESTs by cluster size. The data represent clustering of Centaurea ESTs into unique sequence clusters (unigenes) and show distribution by cluster size. The 4969 Centaurea ESTs were assembled into 4423 unique contigs or 'unigenes' using the PLAN database (Noble foundation). In total, 4075 singlet ESTs were unique (not pictured on graph); 348 could be assembled into clusters containing one or more Centaurea unigene, and were plotted relative to their abundance in the EST library. [file 1471-2229-7-25-S1.doc]

Additional File 1

**Cluster Size**

**(*Centaurea* ESTs per Unigene)**

245

59

23

12

3

3

3

0

50

100

150

200

250

300

2

3

4

5

6

7

more

**Number of ESTs per *Centaurea* Unigene**

**Number**

***Centaurea* Unigenes**

**Distribution of assembled *Centaurea* ESTs by cluster size.** The 4969 *Centaurea* ESTs were assembled into 4423 unique contigs or ‘unigenes’ using the PLAN database (Nobel foundation). In total, 4075 singlet ESTs were unique (not pictured on graph); 348 could be assembled into clusters containing one or more *Centaurea* unigene, and were plotted relative to their abundance in the EST library.
