# Supplementary material for: A first step in understanding an invasive weed through its genes: an EST analysis of invasive Centaurea maculosa
Source: BMC Plant Biol. 2007 May 24;7:25. doi: 10.1186/1471-2229-7-25 (PMC1890287; doi:10.1186/1471-2229-7-25)
Supplement: Additional file 2 — Title: Distribution of Centaurea unigenes by sequence length. The data represent distribution of Centaurea unigenes by sequence length. The 4423 Centaurea unigenes were plotted by their relative abundance based on sequence length in base pairs. [file 1471-2229-7-25-S2.doc]

Additional File 2


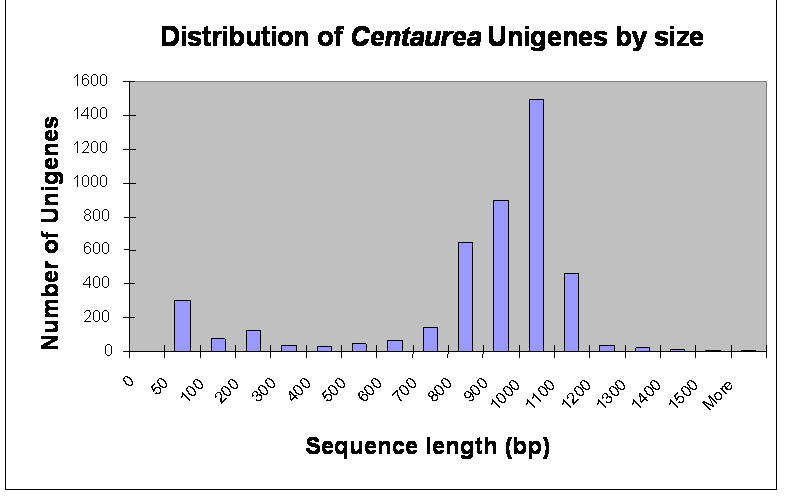


**Distribution of *Centaurea* unigenes by sequence length**. The 4423 *Centaurea* unigenes were plotted by their relative abundance based on sequence length in base pairs.
