# Supplementary material for: A first step in understanding an invasive weed through its genes: an EST analysis of invasive Centaurea maculosa
Source: BMC Plant Biol. 2007 May 24;7:25. doi: 10.1186/1471-2229-7-25 (PMC1890287; doi:10.1186/1471-2229-7-25)
Supplement: Additional file 3 — Taxonomic clades associated with Centaurea unigene top BLAST hits. The data represent taxonomic clades associated with the top similarities of known sequences to Centaurea unigenes. Centaurea unigenes were used to query BLASTX nr database, and the top hit for each unigene was deposited in the PLAN database (3392 unigenes had significant top BLAST hits, others were unable to be annotated). These top hits were assembled by taxonomic group. Approximately 35% of the unigenes had top hits to Arabidopsis, which is part of the Rosid clade. [file 1471-2229-7-25-S3.doc]

Additional File 3


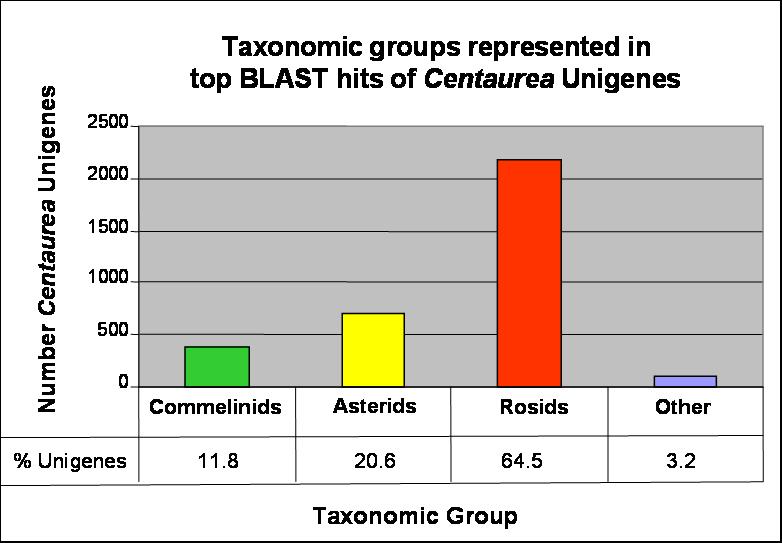


**Taxonomic clades associated with *Centaurea* unigene top BLAST hits**. *Centaurea* unigenes were used to query BLASTX nr database, and the top hit for each unigene was deposited in the PLAN database (3392 unigenes had significant top BLAST hits, others were unable to be annotated). These top hits were assembled by taxonomic group. Approximately 35% of the unigenes had top hits to *Arabidopsis*, which is part of the Rosid clade.
