# Supplementary material for: A first step in understanding an invasive weed through its genes: an EST analysis of invasive Centaurea maculosa
Source: BMC Plant Biol. 2007 May 24;7:25. doi: 10.1186/1471-2229-7-25 (PMC1890287; doi:10.1186/1471-2229-7-25)
Supplement: Additional file 4 — Evolution/Plasticity-related sequences in Centaurea cDNA library. The table lists sequences identified in the Centaurea cDNA library that may be related to evolution and plasticity, based on similarity to known sequences. Evolution-plasticity related sequences from the Centaurea cDNA library are represented by Centaurea unigene identification number (from the PLAN database). Accession number, organism, functional description, and E value of the top BLAST hit for each unigene is listed. [file 1471-2229-7-25-S4.doc]

Additional File 4

**Evolution/Plasticity-related sequences in *Centaurea*** cDNA library

| **Centuarea ID** | **Top BLAST**  **hit ID** | **Organism** | **Function** | **E value** |
| --- | --- | --- | --- | --- |
| **(A) Mobile element-related sequences** | | | | |
| CENT_UG_01296 | AAX92763 | *O. sativa* | transposon protein, putative | 3e-78 |
| CENT_UG_02640 | ABA99201 | *O. sativa* | transposon protein, putative, mutator sub-class (class II) | 7e-21 |
| CENT_UG_00681 | ABB46630 | *O. sativa* | transposon protein, putative, CACTA, En/Spm sub-class (CATCA) | 8e-55 |
| CENT_UG_03112 | BAA22788 | *V. faba* | retrotransposon-like gene (class I) | 2e-06 |
| CENT_UG_03716 | AAX92941 | *O. sativa* | retrotransposon protein, putative, Ty1-copia sub-class (class I) | 8e-13 |
| CENT_UG_01089 | ABA97402 | *O. sativa* | retrotransposon protein, putative, unclassified (class I) | 3e-15 |
| **(B) Heat-shock-related sequences** | | | | |
| CENT_UG_03940 | AAR12194 | *N. benthamiana* | Molecular chaperone Hsp90-2 | 2e-98 |
| CENT_UG_03246 | AAF23074 | *T. aestivum* | heat shock protein 70 | 4e-99 |
| CENT_UG_00169 | AAL38353 | *A. thaliana* | putative heat-shock protein | 1e-82 |
| CENT_UG_01316 | AAM67147 | *A. thaliana* | putative heat shock protein | 2e-38 |
| CENT_UG_01530 | AAN63805 | *P. dulcis* | heat shock protein 60 | 4e-111 |
| CENT_UG_00637 | AAS57912 | *V. radiata* | 70 kDa heat shock cognate protein 1 | 8e-74 |
| CENT_UG_00352 | BAA83710 | *N. tabacum* | heat shock factor | 8e-44 |
| CENT_UG_02194 | CAA47345 | *P. vulgaris* | 70 kDa heat shock protein | 9e-100 |

Evolution/plasticity related sequences from the *Centaurea* cDNA library are represented by *Centaurea* unigene identification number (from the PLAN database). Accession number, organism, functional description, and E value of the top BLAST hit for each unigene is listed.
