# Supplementary material for: A first step in understanding an invasive weed through its genes: an EST analysis of invasive Centaurea maculosa
Source: BMC Plant Biol. 2007 May 24;7:25. doi: 10.1186/1471-2229-7-25 (PMC1890287; doi:10.1186/1471-2229-7-25)
Supplement: Additional file 5 — Alignment of Centaurea unigene (CENT_UG_03500) and related sequences. The data represent predicted amino acid sequence alignment of Centaurea unigene 03500 with related sequences involved in sesquiterpene lactone synthesis. Sesquiterpene lactone synthesis proteins from Artemisia obtusifolia and A. annua were aligned with Centaurea unigene 03500 using (Clustal W). Stars (*) indicate complete sequence conservation;(:) represents amino acids of a similar nature. [file 1471-2229-7-25-S5.doc]

Additional File 5


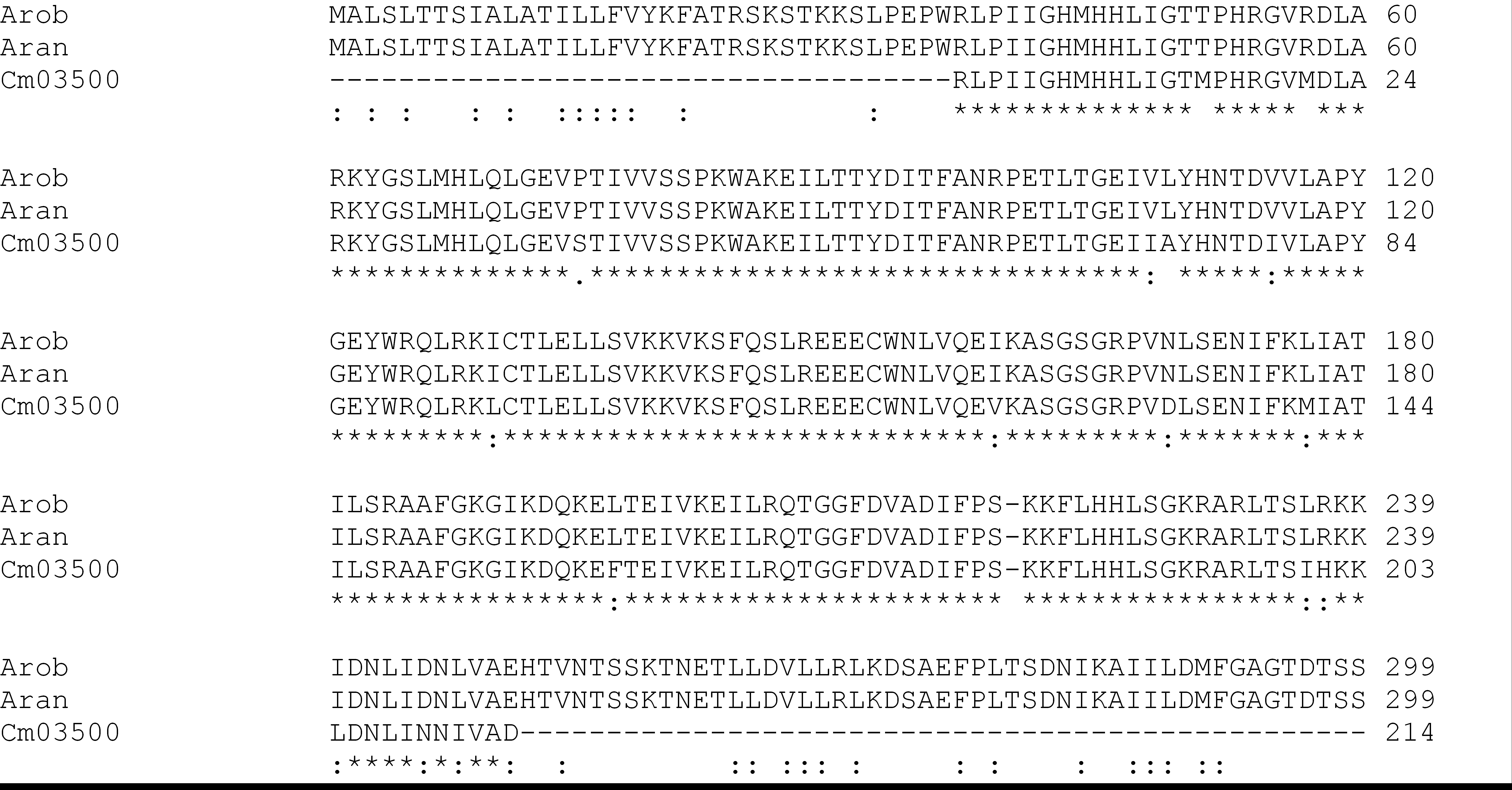


**Alignment of *Centaurea* unigene (CENT_UG_03500) and related sequences**. Sesquiterpene lactone synthesis proteins from *Artemesia obtusifolia* and *A. annua* were aligned with *Centaurea* unigene 03500 using (Clustal W). Stars **(*)** indicate complete sequence conservation; **(:)** represents amino acids of a similar nature.
