# Supplementary material for: A first step in understanding an invasive weed through its genes: an EST analysis of invasive Centaurea maculosa
Source: BMC Plant Biol. 2007 May 24;7:25. doi: 10.1186/1471-2229-7-25 (PMC1890287; doi:10.1186/1471-2229-7-25)
Supplement: Additional file 6 — Flavanoid Pathway- related sequences in Centaurea cDNA library. The table lists sequences identified in the Centaurea cDNA library that may be involved in the flavanoid pathway, based on similarity to known sequences. Proposed function (Func) of flavanoid pathway related sequences in the Centaurea cDNA library; PAL (phenylalanine ammonia lyase), C4H(cinnamate 4-hydroxylase), C4L (4-coumaryl-CoA ligase), CHS (chalcone synthase), CHI (chalcone isomerase), F3'H (flavanoid 3'-hydroxylase), GT (glycosyl transferase), OMT (O-methyltransferase). The number of unigenes and their identification numbers (PLAN database) are listed for each functional group. [file 1471-2229-7-25-S6.doc]

Additional File 6

**Flavanoid Pathway- related sequences in *Centaurea* cDNA library.**

| **Func** | **Unigenes (potenial genes)** | **Unigene IDs** |
| --- | --- | --- |
| PAL | 8 (3) | CENT_UG_00151, CENT_UG_00435, CENT_UG_00996, CENT_UG_01487, CENT_UG_02666, CENT_UG_03772, CENT_UG_04127, CENT_UG_04157 |
| C4H | 1 (1) | CENT_UG_00037 |
| C4L | 1 (1) | CENT_UG_03187 |
| CHS | 5 (3) | CENT_UG_00019, CENT_UG_00766, CENT_UG_01650, CENT_UG_02133, CENT_UG_02557 |
| CHI | 2 (1) | CENT_UG_01866, CENT_UG_01424 |
| F3'H | 5 (3) | CENT_UG_00279, CENT_UG_01543, CENT_UG_02213, CENT_UG_04214, CENT_UG_04285 |
| GT | 11 (7) | CENT_UG_00763, CENT_UG_01670, CENT_UG_01900, CENT_UG_02397, CENT_UG_02287, CENT_UG_02550, CENT_UG_03048, CENT_UG_03142, CENT_UG_02000, CENT_UG_03287, CENT_UG_03943 |
| OMT | 15 (10) | CENT_UG_00334, CENT_UG_00392, CENT_UG_00797, CENT_UG_01026, CENT_UG_01428, CENT_UG_01587, CENT_UG_02428, CENT_UG_02877, CENT_UG_02907, CENT_UG_03027, CENT_UG_03144, CENT_UG_03280, CENT_UG_03376, CENT_UG_03404, CENT_UG_04124 |

Proposed function (Func) of flavanoid pathway related sequences in the *Centaurea* cDNA library; PAL (phenylalanine ammonia lyase), C4H (cinnamate 4-hydroxylase), C4L (4-coumaryl-CoA ligase), CHS (chalcone synthase), CHI (chalcone isomerase), F3'H (flavanoid 3'-hydroxylase), GT (glycosyl transferase), OMT (O-methyltransferase). The number of unigenes and their identification numbers (PLAN database) are listed for each functional group.
