# Supplementary material for: A first step in understanding an invasive weed through its genes: an EST analysis of invasive Centaurea maculosa
Source: BMC Plant Biol. 2007 May 24;7:25. doi: 10.1186/1471-2229-7-25 (PMC1890287; doi:10.1186/1471-2229-7-25)
Supplement: Additional file 7 — Defense-response-related sequences in Centuarea cDNA library. The table lists sequences identified in the Centaurea cDNA library that may be involved in defense response, based on similarity to known sequences. Defense-response-related sequences from the Centaurea cDNA library are represented by Centaurea unigene identification number (PLAN database). Accession number, organism, functional description, and E value of the top BLAST hit for each unigene is listed. [file 1471-2229-7-25-S7.doc]

Additional File 7

**Defense-response-related sequences in *Centaurea*** cDNA library

| **Centaurea ID** | **Top BLAST hit ID** | **Organism** | **Function** | **E value** |
| --- | --- | --- | --- | --- |
| **(A) Defense response-related sequences** | | | | |
| CENT_UG_01551  CENT_UG_02121  CENT_UG_00731 | AT1G55020  AT1G17420.1  AT1G72520.1 | *A. thaliana* | LOX1, lipoxygenase; response to wounding, jasmonic acid biosynthesis, response to abscisic and jasmonic acid stimulus, defense response to pathogenic bacteria, incompatible interaction, growth.  LOX3.  lipoxygenase, putative. | 7e-90  8e-11  9e-90 |
| CENT_UG_04157  CENT_UG_01487  CENT_UG_00996  CENT_UG_02666  CENT_UG_03772  CENT_UG_04127  CENT_UG_00435  CENT_UG_00151 | AT2G37040.1  AT3G53260.1 | *A. thaliana* | Phenylalanine ammonia-lyase 1 (PAL1); defense response, response to wounding, phenylalanine ammonia-lyase activity.  PAL2 | 2e-116  3e-116  2e-113  7e-108  4e-102  2e-24  2e-108  4e-81 |
| CENT_UG_00144  CENT_UG_03728  CENT_UG_00973  CENT_UG_03454  CENT_UG_01864 | AT4G02600.1  AT5G65970.1  NP_851188  (AT5G54250) | *A. thaliana* | Seven trans-membrane MLO family protein; calmodulin binding, defense response, cell death.  Calmodulin binding, defense response, cell death.  ATCNGC4 (DEFENSE, NO DEATH 2, DND2); calmodulin binding, cation channel, cyclic nucleotide binding. | 1e-99  2e-46  2e-110  4e-62  2e-76 |
| CENT_UG_01096  CENT_UG_00932  CENT_UG_01174  CENT_UG_03330 | AT3G20820.1 | *A. thaliana* | Leucine-rich repeat, plant specific; protein binding, signal transduction, defense response. | 4e-84  1e-78  5e-76  2e-74 |
| CENT_UG_00611 | AT3G48090.1 | *A. thaliana* | Disease resistance protein (EDS1); signal transducer activity, triacylglycerol lipase activity, lipid metabolism, defense response. | 4e-22 |
| CENT_UG_00558 | AT3G55230.1 | *A. thaliana* | Disease resistance-responsive family protein; molecular function unknown, defense response. | 8e-10 |
| CENT_UG_03760  CENT_UG_02198  CENT_UG_01724 | AT5G47910.1 | *A. thaliana* | Respiratory burst oxidase protein D (RbohD);  oxygen and reactive oxygen species metabolism, defense response | 2e-110  1e-65  2e-59 |
| CENT_UG_00851  CENT_UG_04111 | AT1G58170.1 | *A. thaliana* | Disease resistance-responsive protein-related / dirigent protein-related; molecular function unknown, defense response, lignan biosynthesis. | 5e-54  1e-53 |
| CENT_UG_02248  CENT_UG_00531  CENT_UG_00565 | AT5G04720.1 | *A. thaliana* | Disease resistance protein (CC-NBS-LRR class), putative; ADR1-L2, ADR1-LIKE 2, defense response. | 3e-65  4e-65  4e-65 |
| CENT_UG_00867  CENT_UG_00804 | CAA87071 | *S. nigra* | Pathogenesis-related protein, PR-1 type | 5e-57  5e-44 |
| CENT_UG_02248 | ABA99732 | *O. sativa* | Disease resistance, putative | 1e-71 |
| CENT_UG_00565 | Q9SZA7, AT4G33300 | *A. thaliana* | Probable disease resistance protein | 3e-65 |
| **(B) LRR and leucine zipper domain containing sequences** | | | | |
| CENT_UG_04303 | AAL12626 | *A. thaliana* | leucine-rich repeat receptor-like kinase F21M12.36 | 7e-71 |
| CENT_UG_04152 | AAM65656 | *A. thaliana* | leucine rich repeat protein, putative | 1e-88 |
| CENT_UG_01096  CENT_UG_01174 | CAE76632 | *Cicer arietinum* | leucine rich repeat protein | 3e-87  2e-76 |
| CENT_UG_01558 | NP_922826 | *O. sativa* | putative leucine-rich repeat protein | 2e-26 |
| CENT_UG_02258 | BAC22512 | *Z. elegans* | homeobox leucine-zipper protein | 9e-101 |
| CENT_UG_04221 | CAA11499 | *S. oleracea* | basic leucine zipper protein | 2e-20 |
| CENT_UG_01937 | CAA64221 | *P. brachycarpa* | homeobox-leucine zipper protein | 1e-14 |
| **(C) WRKY transcription factors** | | | | |
| CENT_UG_02392 | AAW67002 | *C. annuum* | WRKY transcription factor-c | 1e-16 |
| CENT_UG_02512 | AAZ99027 | *C. annuum* | WRKY-A1244 | 5e-11 |
| CENT_UG_03094 | BAB16432 | *N. tabacum* | WRKY transcription factor NtEIG-D48 | 7e-07 |
| CENT_UG_03349 | BAE46417 | *S. tuberosum* | Double WRKY type transfactor | 1e-64 |
| CENT_UG_02998 | AAC49528 | *P. crispum* | WRKY3 | 1e-05 |
| CENT_UG_04064 | AAC31956 | *P. brachycarpa* | Zince finger protein WRKY1 | 7e-28 |
| CENT_UG_03882  CENT_UG_00878 | NP_564792 | *A. thaliana* | WRKY6; transcription factor | 3e-61  4e-56 |
| CENT_UG_00803 | NP_179913 | *A. thanliana* | WRKY15; transcription factor | 7e-08 |
| CENT_UG_01139 | NP_192034 | *A. thaliana* | WRKY22; transcription factor | 3e-42 |
| CENT_UG_02403 | NP_192845 | *A. thaliana* | WRKY41; transcription factor | 5e-26 |
| CENT_UG_00385 | NP_192354 | *A. thalinaa* | WRKY42; transcription factor | 6e-46 |

Defense-response-related sequences from the *Centaurea* cDNA library are represented by *Centaurea* unigene identification number (PLAN database). Accession number, organism, functional description, and E value of the top BLAST hit for each unigene is listed.
